# Defining the mechanism of galectin-3–mediated TGF-β1 activation and its role in lung fibrosis

**DOI:** 10.1016/j.jbc.2024.107300

**Published:** 2024-04-18

**Authors:** Jessica F. Calver, Nimesh R. Parmar, Gemma Harris, Ryan M. Lithgo, Panayiota Stylianou, Fredrik R. Zetterberg, Bibek Gooptu, Alison C. Mackinnon, Stephen B. Carr, Lee A. Borthwick, David J. Scott, Iain D. Stewart, Robert J. Slack, R. Gisli Jenkins, Alison E. John

**Affiliations:** 1 School of Medicine, University of Nottingham, Nottingham, United Kingdom; 2 Stevenage Bioscience Catalyst, Galecto Biotech AB, Stevenage, United Kingdom; 3 Roche Products Limited, Welwyn Garden City, Hertfordshire, United Kingdom; 4 Research Complex at Harwell, Rutherford Appleton Laboratory, Didcot, Oxfordshire, United Kingdom; 5 School of Biosciences, University of Nottingham, Loughborough, Leicestershire, United Kingdom; 6 Membrane Protein Laboratory, Diamond Light Source, Rutherford Appleton Laboratory, Didcot, Oxfordshire, United Kingdom; 7 Diamond Light Source, Diamond House, Rutherford Appleton Laboratories, Didcot, Oxfordshire, United Kingdom; 8 Institute for Lung Health, NIHR Leicester Respiratory Biomedical Research Centre, Glenfield Hospital, Leicester, United Kingdom; 9 Leicester Institute for Structural and Chemical Biology, Henry Wellcome Building, University of Leicester, Leicester, United Kingdom; 10 Galecto Biotech AB, Sahlgrenska Science Park, Gothenburg, Sweden; 11 Galecto Biotech AB, Nine Edinburgh BioQuarter, Edinburgh, United Kingdom; 12 Department of Chemistry, University of Oxford, Oxford, Oxfordshire, United Kingdom; 13 Fibrofind Ltd, Newcastle upon Tyne, United Kingdom; 14 Newcastle Fibrosis Research Group, Biosciences Institute, Newcastle University, Newcastle upon Tyne, United Kingdom; 15 National Heart and Lung Institute, Imperial College London, London, United Kingdom

**Keywords:** pulmonary fibrosis, fibroblast, transforming growth factor beta (TGF-β), integrin, galectin

## Abstract

Integrin-mediated activation of the profibrotic mediator transforming growth factor-β1 (TGF-β1), plays a critical role in idiopathic pulmonary fibrosis (IPF) pathogenesis. Galectin-3 is believed to contribute to the pathological wound healing seen in IPF, although its mechanism of action is not precisely defined. We hypothesized that galectin-3 potentiates TGF-β1 activation and/or signaling in the lung to promote fibrogenesis. We show that galectin-3 induces TGF-β1 activation in human lung fibroblasts (HLFs) and specifically that extracellular galectin-3 promotes oleoyl-L-α-lysophosphatidic acid sodium salt–induced integrin-mediated TGF-β1 activation. Surface plasmon resonance analysis confirmed that galectin-3 binds to αv integrins, αvβ1, αvβ5, and αvβ6, and to the TGFβRII subunit in a glycosylation-dependent manner. This binding is heterogeneous and not a 1:1 binding stoichiometry. Binding interactions were blocked by small molecule inhibitors of galectin-3, which target the carbohydrate recognition domain. Galectin-3 binding to β1 integrin was validated *in vitro* by coimmunoprecipitation in HLFs. Proximity ligation assays indicated that galectin-3 and β1 integrin colocalize closely (≤40 nm) on the cell surface and that colocalization is increased by TGF-β1 treatment and blocked by galectin-3 inhibitors. In the absence of TGF-β1 stimulation, colocalization was detectable only in HLFs from IPF patients, suggesting the proteins are inherently more closely associated in the disease state. Galectin-3 inhibitor treatment of precision cut lung slices from IPF patients’ reduced Col1a1, TIMP1, and hyaluronan secretion to a similar degree as TGF-β type I receptor inhibitor. These data suggest that galectin-3 promotes TGF-β1 signaling and may induce fibrogenesis by interacting directly with components of the TGF-β1 signaling cascade.

Idiopathic pulmonary fibrosis (IPF) is a chronic and progressive interstitial lung disease (1, 2). It is the most common interstitial lung disease subtype with over 32,000 patients living with the disease in the United Kingdom and more than 6000 newly diagnosed cases annually (3). Older adults and males are primarily affected, with a median survival of 2.5 to 3.5 years from time of diagnosis (1, 4, 5). Repeated injury to the distal lung parenchyma of a genetically predisposed individual promotes apoptotic, necrotic, and senescent pathways, causing alveolar collapse and exposure of the underlying basement membrane to damage (6, 7, 8, 9). Compromised re-epithelialization following injury enables interstitial cells to proliferate and induces downstream signaling pathways, promoting progressive lung fibrosis (10). Currently, there is no cure for IPF and only two antifibrotic drugs, pirfenidone and nintedanib, are licensed for IPF management (11, 12, 13).

Transforming growth factor-β1 (TGF-β1) is a potent profibrotic mediator that is upregulated in IPF patients, with its protein expression concentrated to fibroblastic foci and associated with sites of active fibrosis and collagen biosynthesis (14, 15, 16). Activation of TGF-β1 *via* αv integrins is central to fibrogenesis, with both genetic models using integrin β1−/− and integrin β6−/− mice as well as anti-β6 mAbs ameliorating bleomycin-induced lung fibrosis (17, 18, 19, 20, 21). Similarly in patients with IPF inhibition of αvβ6 showed promise in phase II clinical trials, although the mAb, BG00011 (formerly STX-100) demonstrated substantial toxicity limiting its efficacy (22, 23). Therefore, alternative strategies to inhibit TGF-β1 activation and signaling are required.

Galectin-3 is a chimeric and self-associating, profibrotic beta-galactoside–binding protein localized both intracellularly and extracellularly that is believed to contribute to the pathological wound healing seen in IPF individuals (24, 25, 26, 27). Usual interstitial pneumonia patient lung sections immunostained for galectin-3 show elevated galectin-3 expression within fibroblastic foci that is temporospatial associated with fibrosis (28). Galectin-3 levels are also higher in the serum and bronchoalveolar lavage fluid of IPF patients than controls (28, 29). Following irradiation-induced lung injury *in vivo*, galectin-3 is predominately secreted by alveolar macrophages and its expression markedly increased in fibrotic lung tissue (30). Additionally, the distribution of galectin-3 is spatially related to fibrotic regions of the lung following administration of adenoviral TGF-β1 or intratracheal bleomycin *in vivo* and its mitogenic activity on cultured human lung fibroblasts (HLFs) has also been demonstrated (28, 31). Galectin-3 significantly induces fibroblast migration and increases fibroblast collagen synthesis *in vitro* further suggesting a role for galectin-3 in fibrogenesis (29). In murine models of pulmonary fibrosis, loss of galectin-3 is protective, with galectin-3−/− mice demonstrating decreased collagen staining and lower total lung collagen compared with WT mice (28). Adding to this, the novel small-molecule inhaled galectin-3 inhibitor, GB0139 (formerly TD139), designed to modulate the fibrogenic response to tissue injury has been shown to decrease galectin-3 expression in the lung and significantly reduce bleomycin-induced fibrosis as assessed by total lung collagen content (28). In a phase 1/2a clinical trial (ID: NCT02257177), inhalation of GB0139 was shown highly suitable for dosing IPF patients and was associated with reductions in plasma biomarkers linked to IPF pathobiology, including platelet-derived growth factor-beta, plasminogen activator inhibitor-1, galectin-3, chemokine (C-C motif) ligand 18, and chitinase-3–like protein 1 (YKL-40) (32). GB0139 has since progressed into a European Union and US phase 2b clinical trial in IPF subjects “GALACTIC-1” (ID: NCT03832946) with the full readout yet to the published.

Galectin-3 preferentially binds to N-glycan residues, which are covalently linked to the TGF-β1 receptor during posttranslation processing. Galectin-3 has been proposed to mediate cell surface retention of the TGF-β1 receptor by binding to its poly-N-acetyllactosamine residues, potentially sustaining profibrotic signaling (28, 33). Similar to the TGF-β1 receptor, the literature demonstrates that galectin-3 is also capable of regulating integrin activity by interacting with their surface glycans (34, 35, 36, 37). Therefore, we sought to define the mechanism through which galectin-3 may enhance TGF-β1 activation and contribute to the development of IPF.

## Results

### Galectin-3 induces TGF-β1 activation in fibroblasts but not epithelial cells

**Figure 1:**
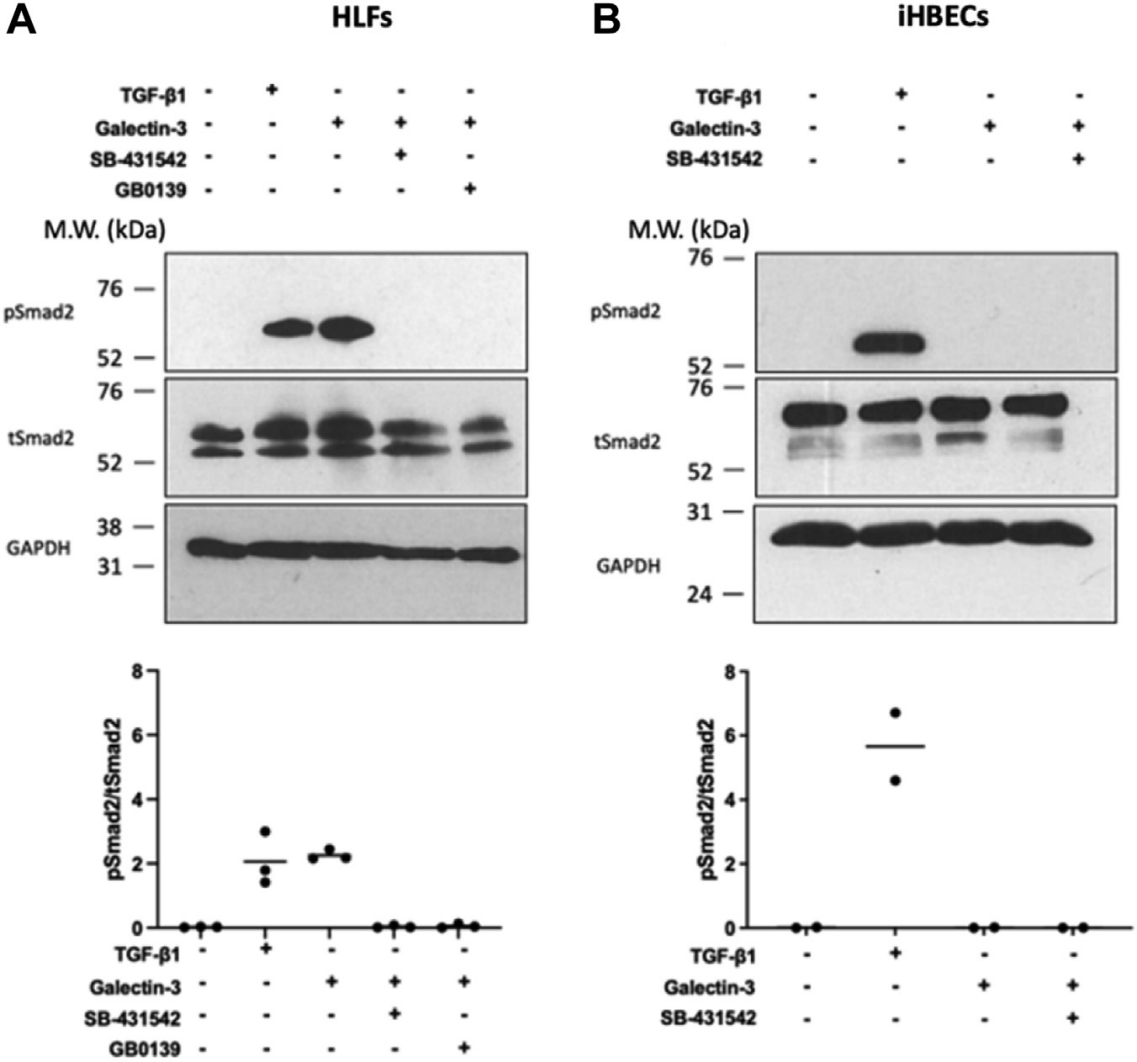
**Galectin-3 induces TGF-β1 activation in fibroblasts.** Representative Western blot of pSmad2 levels in (*A*) non-IPF HLFs (N = 3) and (*B*) iHBECs (N = 2) pretreated with 50 μM SB-431542 (ALK5 inhibitor) or 1 μM GB0139 (galectin-3 inhibitor) for 20 min prior to 2-h treatment with 10 μg/ml galectin-3 or 2 ng/ml TGF-β1. Western blot bands were quantified using densitometry analysis and presented as a ratio of pSmad2/tSmad2. ALK, activin receptor–like kinase; HLF, human lung fibroblast; iHBEC, immortalized human bronchial epithelial cell line; IPF, idiopathic pulmonary fibrosis.; TGF-β1, transforming growth factor-β1.

To investigate whether galectin-3 was able to induce TGF-β1 activation, primary HLFs and an immortalized human bronchial epithelial cell line (iHBECs) were treated with exogenous galectin-3 protein and Smad2 phosphorylation was assessed by Western blotting. Although treatment with galectin-3 induced Smad2 phosphorylation in HLFs (Fig. 1*A*), it was unable to promote phosphorylation of Smad2 in the epithelial cell line (Fig. 1*B*). In contrast, direct stimulation with TGF-β1 was shown to induce Smad2 phosphorylation in both cell types (Fig. 1, *A* and *B*). Galectin-3–induced Smad2 phosphorylation in HLFs was completely inhibited by pretreatment either with the high affinity galectin-3 inhibitor, GB0139, or with the selective inhibitor of the TGF-β type I receptor, activin receptor–like kinase 5 (ALK5) SB-431542 (Fig. 1*A*).

### LPA-induced integrin-mediated TGF-β1 activation requires galectin-3

**Figure 2:**
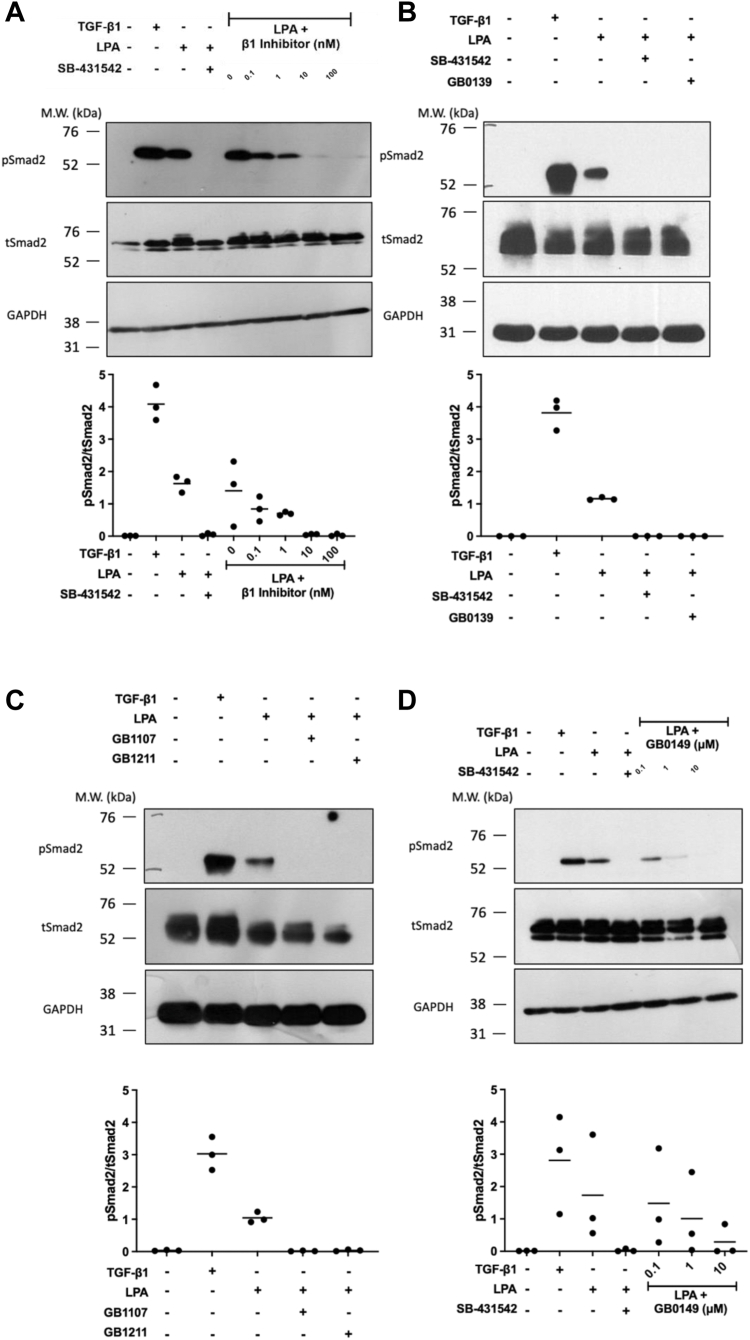
**Integrin-mediated TGF-β1 activation requires extracellular galectin-3.** Representative Western blots of pSmad2 levels in non-IPF HLFs (N = 3) pre-treated with (*A*) NOTT199SS β1 inhibitor (0.1–100 nM) or (*B*–*D*) galectin-3 inhibitors GB0139, GB1107, and GB1211 (1 μM) or GB0149 (0.1–10 μM) for 20 min prior to stimulation with 2 ng/ml TGF-β1 (2-h) or 50 μM LPA (4-h). Cells pretreated with 50 μM SB-431542 (ALK5 inhibitor) were included as a control demonstrating maximal inhibition of pSmad2 signaling. Western blot bands were quantified using densitometry analysis and presented as a ratio of pSmad2/tSmad2. ALK, activin receptor–like kinase; HLF, human lung fibroblast; iHBEC, immortalized human bronchial epithelial cell line; IPF, idiopathic pulmonary fibrosis; LPA, lysophosphatidic acid; TGF-β1, transforming growth factor-β1.

To investigate the potential involvement of galectin-3 in promoting integrin-mediated TGF-β1 activation in fibroblasts using galectin-3 inhibitors with varying K_d_ values and cell permeability (Table S1), HLFs were stimulated with oleoyl-L-α-lysophosphatidic acid sodium salt (LPA), a G-protein agonist, which promotes reorganization of the cytoskeleton and integrin activation in a variety of cell types. LPA-induced TGF-β1 activation in HLFs as measured by Smad2 phosphorylation was inhibited in a concentration-dependent manner by pretreatment of HLFs with the β1 integrin small molecule inhibitor, NOTT199SS (Fig. 2*A*). LPA-induced Smad2 phosphorylation in HLFs was also inhibited by pretreatment both with the inhaled galectin-3 inhibitor, GB0139 (Fig. 2*B*), and by the orally active galectin-3 inhibitors, GB1107 and GB1211 (Fig. 2*C*). Pretreatment with the cell-impermeable extracellular galectin-3 inhibitor, GB0149 (38) also inhibited phosphorylation of Smad2 in a concentration-dependent manner (Fig. 2*D*), suggesting that galectin-3 might be mediating its effects on TGF-β1 activation extracellularly.

### Galectin-3 interacts physically with integrins αvβ1, αvβ5, and αvβ6 and the TGFβRII subunit

**Figure 3:**
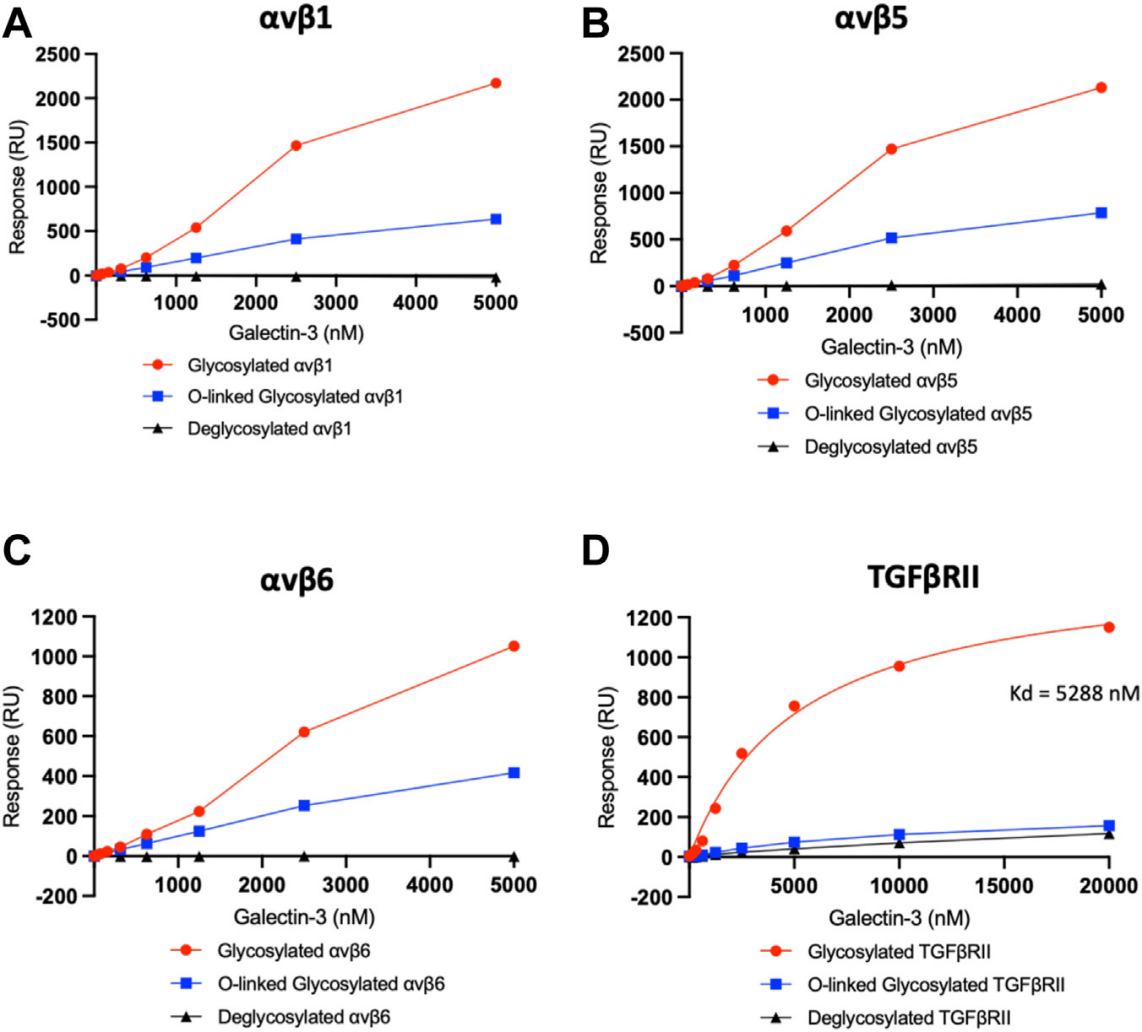
**Galectin-3 binds to αv integrins and TGFβRII in a glycolsylation-dependent manner.** Soluble galectin-3 (sequential injections, 19.5–5000 nM) binding to glycosylated or deglycosylated αv integrins: (*A*) αvβ1, (*B*) αvβ5, and (*C*) αvβ6 immobilized on the surface of a Series S sensor chip CM5 (approximately 1000 RU). *D*, soluble galectin-3 (sequential injections, 156.3–20000 nM) binding to glycosylated or deglycosylated TGFβRII immobilized to a Series S sensor chip CM5 (approximately 400 RU). SPR signals were measured in RU and all sensorgrams baseline-corrected. Binding response values plotted in GraphPad Prism with connecting *line/curve* shown. RU, response units; SPR, surface plasmon resonance.

To determine how galectin-3 promotes integrin-mediated TGF-β1 activation, protein interaction studies were performed by surface plasmon resonance (SPR). Galectin-3 bound to recombinant human integrins αvβ1 (Fig. 3*A*), αvβ5 (Fig. 3*B*), and αvβ6 (Fig. 3*C*) in a concentration-dependent manner. Binding responses were 2-fold higher with αvβ1 and αvβ5 than αvβ6 (Fig. 3, *A*–*C*). The highest binding responses recorded for all three integrins were considerably higher than the theoretical maximum response (approximately 135 Rmax) for analyte-ligand binding 1:1. It was not possible to saturate the immobilized integrins at the galectin-3 concentrations tested, as evidenced by the lack of a plateau. As it was not possible to yield an accurate K_d_ value or Bmax for these binding interactions, the minimum number of individual galectin-3 proteins involved in the binding interactions were calculated: 16, 16, and 8 for αvβ1, αvβ5, and αvβ6, respectively. The binding of galectin-3 to all three integrins (red lines) was seen to be glycosylation-dependent, as enzymatic removal of all N-linked and common O-linked glycans with protein deglycosylation mix II completely inhibited these binding interactions (black lines) (Fig. 3, *A*–*C*). Treatment with PNGase F digestion to remove N-linked glycans alone resulted in only a partial decrease in integrin-galectin-3 binding (blue lines) (Fig. 3, *A*–*C*).

Galectin-3 also bound to the extracellular domain of the recombinant human TGFβRII protein in a concentration-dependent manner (Fig. 3*D*). As with the αv integrins, the highest binding response recorded was higher than the theoretical maximum response (approximately 251 Rmax) for analyte-ligand binding 1:1. As a higher analyte concentration was used for the TGFβRII experiments than for the αv integrins, saturation of the TGFβRII subunit was reached, as evidenced by the plateauing of the curve. Subsequent nonlinear regression analysis yielded a K_d_ value of 5288 nM and a Bmax of 1473 response units (RU), and it was calculated that a minimum of six individual galectin-3 proteins were involved in the binding interaction. Galectin-3 binding to TGFβRII (red line) was also shown to be glycosylation-dependent with enzymatic removal of all N-linked and common O-linked glycans inhibiting the binding interaction (black line) (Fig. 3*D*). However, a similar decrease in the level of binding was observed after the removal of only N-linked glycans (blue line) (Fig. 3*D*), suggesting that the galectin-3–TGFβRII interaction is predominantly N-linked glycan-dependent.

### Galectin-3 inhibitors prevent galectin-3 binding to αv integrins and the TGFβRII subunit

**Figure 4:**
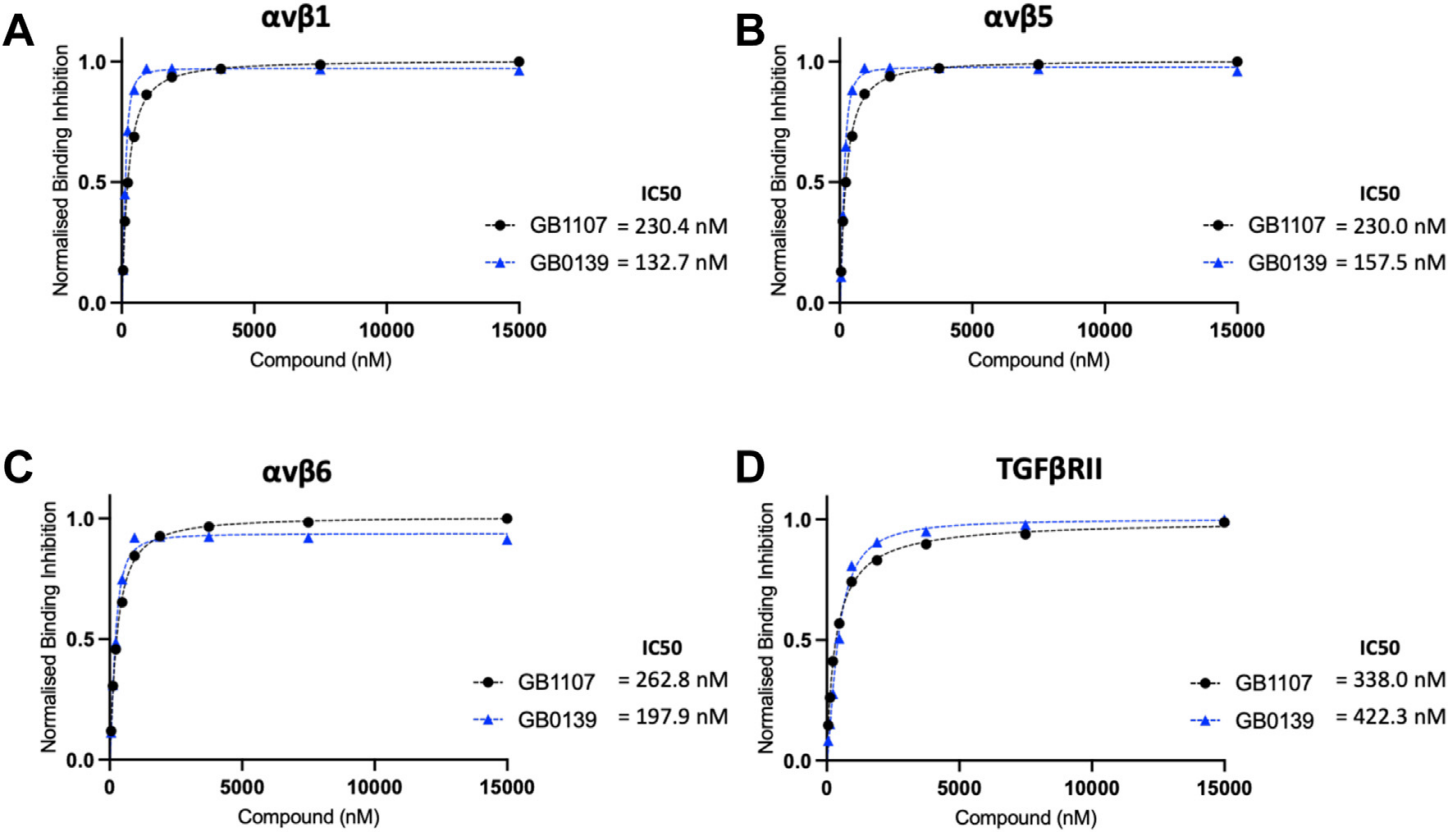
**Galectin-3 inhibitors block αv integrin and TGFβRII binding to galectin-3.** Solution competition binding assays performed with the galectin-3 inhibitor GB0139 (*blue*) or GB1107 (*black*) for αv integrins: (*A*) αvβ1, (*B*) αvβ5, and (*C*) αvβ6 or (*D*) TGFβRII in the presence of galectin-3 at 625 nM. Response values are normalized with respect to the highest binding response (DMSO control) and competitive inhibition graphs plotted in GraphPad Prism. IC_50_ values were calculated by nonlinear regression analysis (binding saturation), and specific binding was calculated with hill slope. DMSO, dimethyl sulfoxide; TGF, transforming growth factor.

To further understand the binding of galectin-3 to integrins and the TGFβRII subunit and the potential to disrupt this interaction with galectin-3 inhibitors, solution competition binding assays were performed. Target inhibition of the galectin-3 carbohydrate recognition domain (CRD) with GB0139 (blue line) or GB1107 (black line) inhibited the binding of galectin-3 to αv integrins: αvβ1 (Fig. 4*A*), αvβ5 (Fig. 4*B*), and αvβ6 (Fig. 4*C*) and the TGFβRII subunit (Fig. 4*D*) in a concentration-dependent manner. These data confirm that the interaction of galectin-3 with both the integrins and TGFβRII are CRD-dependent.

### Galectin-3 coimmunoprecipitates with β1 integrin in HLFs

**Figure 5:**
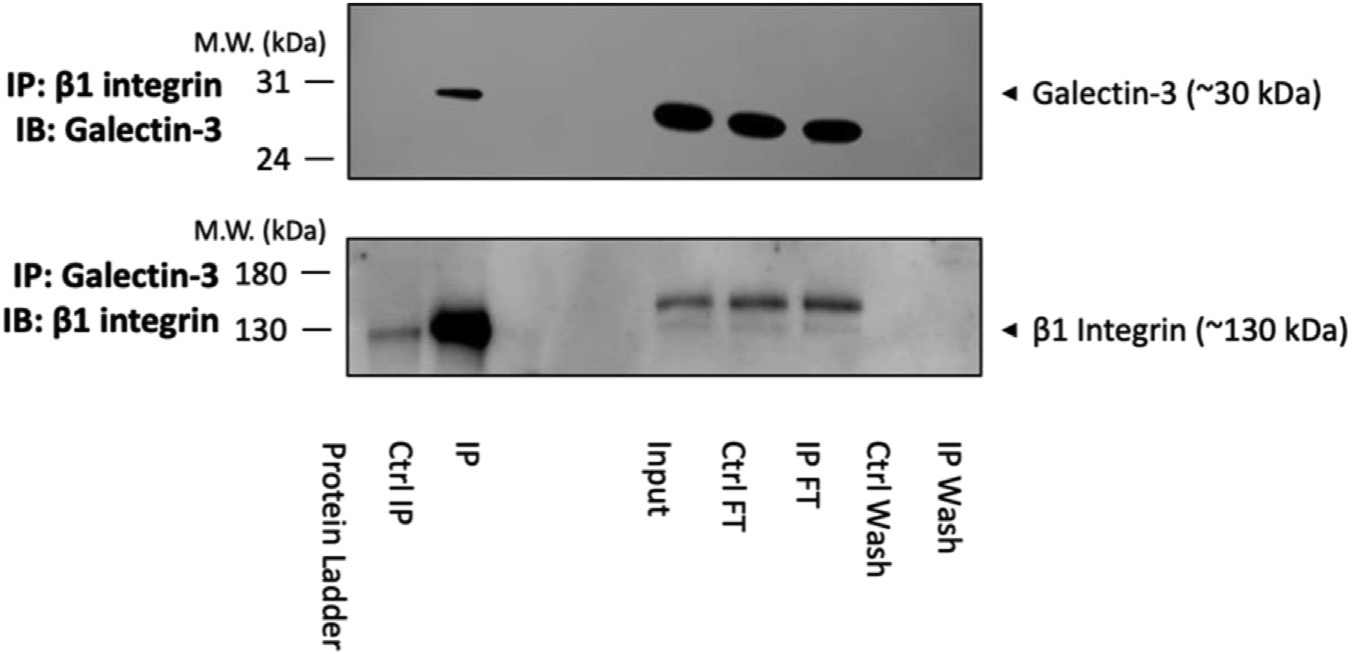
**Binding of galectin-3 to β1 integrin is detected in lung fibroblasts.** Representative Western blots showing coimmunoprecipitation of galectin-3 and the β1 integrin. Whole-cell protein lysates (650 μg/IP reaction) from untreated non-IPF HLFs p6 (N = 3) were immunoprecipitated with an anti-β1 integrin antibody (10 μg/IP reaction) and immunoblotted for galectin-3 (*upper panel*) or immunoprecipitated with an anti-galectin-3 antibody (10 μg/IP reaction) and immunoblotted for the β1 integrin (*lower panel*). Co-IP input, FT, and wash steps loaded as controls. Proteins separated by reducing SDS-PAGE and target protein size estimated from the marker migration pattern. Co-IP, coimmunoprecipitation; FT, flow through; IP, immunoprecipitation; IPF, idiopathic pulmonary fibrosis; HLF, human lung fibroblast.

In order to confirm the key SPR binding data in a cell-based system, coimmunoprecipitation (Co-IP) experiments were performed to determine whether galectin-3 is a β1 integrin-binding partner in HLFs (Fig. 5). Galectin-3 (30 kDa) was successfully coimmunoprecipitated with the β1 integrin, following pulldown (immunoprecipitation [IP]) with a β1 integrin antibody and immunoblot detection with a galectin-3–specific antibody (upper panel). No galectin-3 band was detectable in the sample immunoprecipitated with an IgG control antibody (upper panel). As expected, galectin-3 protein was detected in all input and flow through (FT) control samples (upper panel). For further confirmation of the galectin-3 and β1 integrin interaction, the Co-IP experiment was also performed in the opposite direction (lower panel). The β1 integrin (130 kDa) was successfully coimmunoprecipitated following pulldown with a galectin-3 antibody, although a low level of nonspecific binding was detected in the IgG control IP (lower panel). The β1 integrin was detected in all input and FT controls (lower panel).

### Galectin-3 and β1 integrin are in close proximity in IPF HLFs

**Figure 6:**
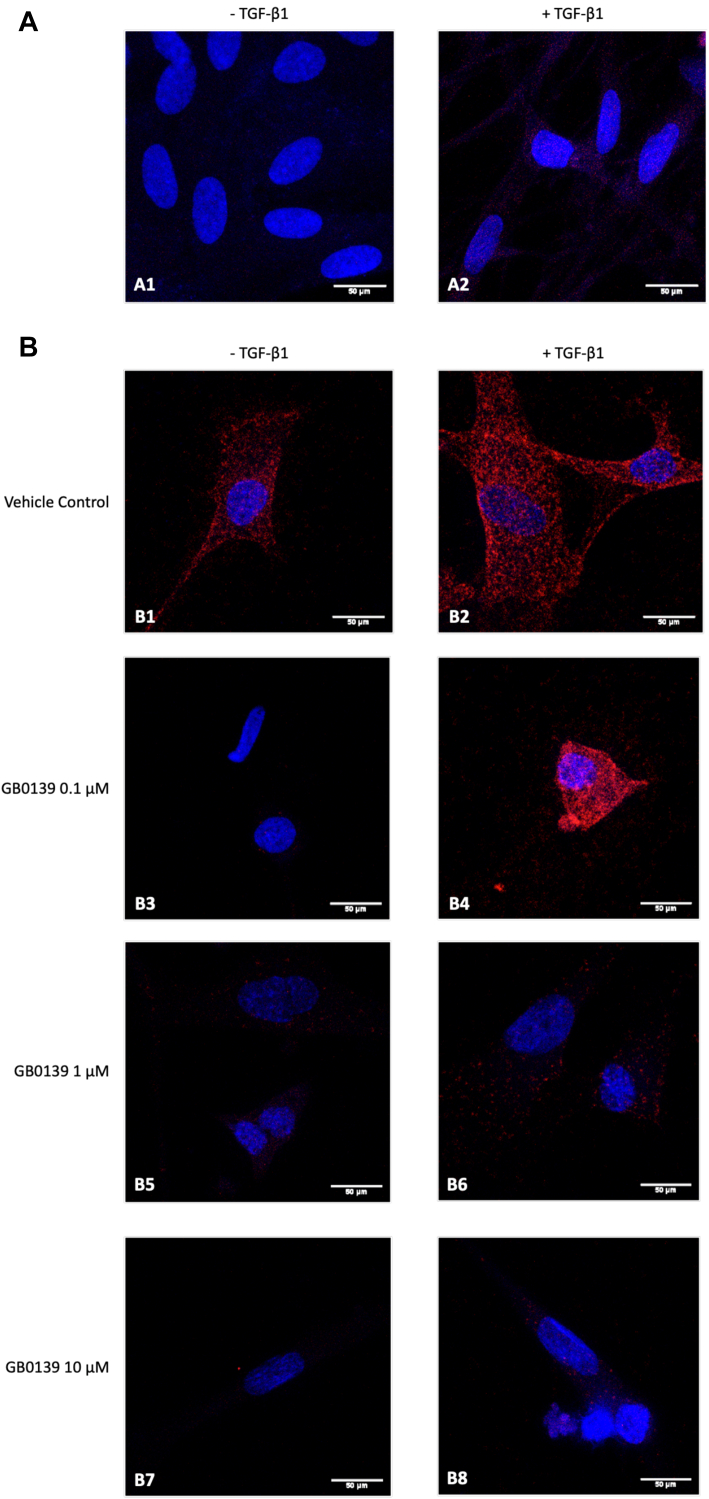
**Galectin-3 and β1 integrin colocalize on the cell surface.** Representative confocal microscopy images (63× magnification) showing PLA of galectin-3 and the β1 integrin in (*A*) non-IPF HLFs p3-4 (N = 3) or (*B*) IPF HLFs p3 (N = 4) in the absence or presence of TGF-β1 stimulation (2 ng/ml TGF-β1 for 24 h) and galectin-3 inhibitor (0.1–10 μM). Cells probed with a mouse anti-β1 integrin primary antibody (5 μg/ml) and a rabbit anti-galectin-3 primary antibody (5 μg/ml), followed by anti-rabbit PLUS and anti-mouse MINUS probes. Colocalization of galectin-3 and the β1 integrin ≤40 nm indicated by *red fluorescence* with 4′,6-diamidino-2-phenylindole counterstaining (*blue*). HLF, human lung fibroblast; IPF, idiopathic pulmonary fibrosis; PLA, proximity ligation assay; TGF-β1, transforming growth factor-β1.

To further confirm that galectin-3 and the β1 integrin colocalize closely on the cell surface of HLFs, a proximity ligation assay (PLA) was performed in both non-IPF (Fig. 6*A*) and IPF (Fig. 6*B*) HLFs. In these experiments, the punctate red fluorescent signal visible in the *z*-stack images is a measure of colocalization events in which galectin-3 and the β1 integrin protein were found within 40 nm of each other within the same cellular compartment. The use of fixed and unpermeabilized cells is consistent with detection of PLA signal only at the cell surface, as it is the effect of treatment with the small molecule GB0139 which shows low cell permeability at the time points used in these experiments. No PLA signal was observed in untreated non-IPF HLFs (panel A1), while stimulation of the non-IPF HLFs with TGF-β1 for 24 h resulted in the appearance of a low-level red fluorescence signal (panel A2). In HLFs isolated from fibrotic lung tissue, higher levels of PLA signals were readily apparent. This was observed in untreated IPF HLFs (panel B1) and was augmented further by stimulation with TGF-β1 (panel B2). To confirm the previous SPR and Co-IP data suggesting that compounds which target the galectin-3 CRD could prevent the interaction of galectin-3 with integrins, PLA studies were also performed in the presence of GB0139 (0.1–10 μM). Pretreatment of the unstimulated and TGF-β1 stimulated IPF HLFs with GB0139 reduced the level of galectin-3 and β1 integrin colocalization in a concentration-dependent manner (panels B1, B3, B5, B7 for unstimulated cells and panels B2, B4, B6, B8 for cells stimulated with TGF-β1). There was abrogation of PLA signal at 1 μM and 10 μM compared to the vehicle control in both stimulated and unstimulated cells. This was also apparent in the unstimulated HLFs at 0.1 μM, but in the stimulated cells, it was not clearly appreciable at this inhibitor concentration, relative to the high expression levels in the vehicle control.

### *Ex vivo* human IPF precision cut lung slice studies

**Figure 7:**
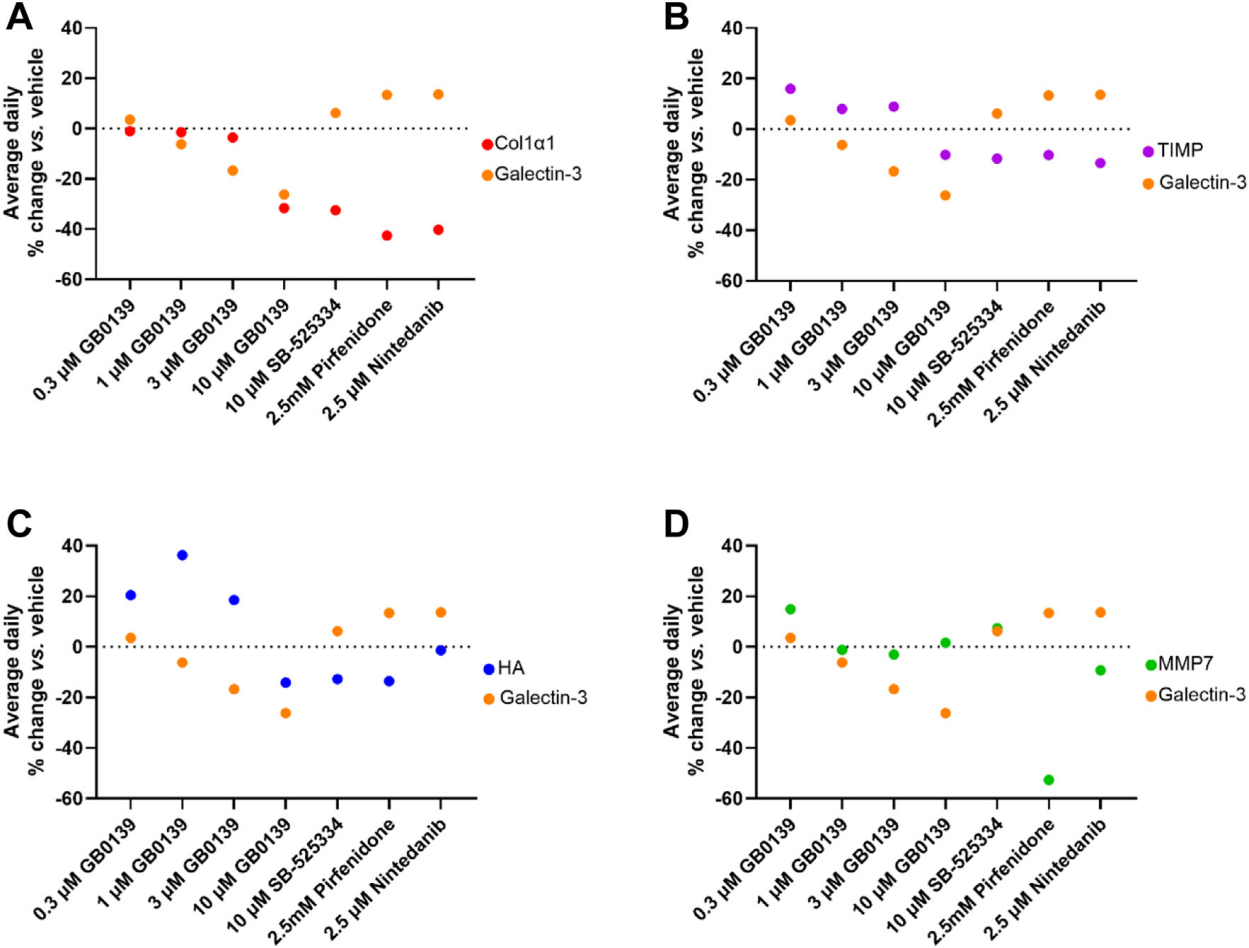
**Galectin-3 inhibitors reduce secretion of fibrotic markers in IPF lung tissue.** The daily average change in the level of fibrotic markers secreted by IPF patient–derived PCLuS was measured in response to treatment with inhibitor. PCLuS were treated with galectin-3 inhibitor GB0139 (0.3–10 μM), 10 μM SB-525334, 2.5 mM pirfenidone, or 2.5 μM nintedanib for 4 days with media replaced every 24 h. Levels of secreted galectin-3, Col1a1 (*A*), TIMP1 (*B*), hyaluronan (*C*), and MMP7 (*D*) were measured daily, and the average value for each mediator (N = 3 donors) is presented as % change in drug treated samples compared with vehicle control. Comparative levels of galectin-3 are shown in each graph. IPF, idiopathic pulmonary fibrosis; PCLuS, precision cut lung slice.

The antifibrotic effect of blocking the interactions of galectin-3 with integrins and TGFbRII with GB0139 was investigated *ex vivo* by measurement of soluble fibrogenesis markers (Co1α1, TIMP-1, MMP-7, galectin-3, hyaluronan in precision cut lung slices (PCLuSs) derived from IPF lung explants. Treatment with GB0139 caused a concentration-dependent reduction in secreted galectin-3 compared to vehicle, yet no effect on galectin-3 was observed with control drugs SB-525334 (TGF-β type I receptor inhibitor), pirfenidone, or nintedanib (Fig. 7). The reduction in galectin-3 levels was associated with a reduction in markers of fibrosis including Co1α1, TIMP-1, and hyaluronan but not on MMP7 and at the highest concentration of GB0139 tested (10 μM) effects were comparable to that observed with SB-525334 and with currently approved IPF therapies, pirfenidone, and nintedanib (Fig. 7). In addition, following treatment of PCLuS with 10 μM GB0139 for 4 days, proteomic analysis confirmed that several signaling pathways were downregulated and these included proteins associated with canonical TGF-β1 Smad signaling (transcriptional activity of Smad2/Smad3:Smad4) in each of the IPF patient–derived PCLuS indicating that galectin-3 inhibition reduces downstream signaling from the TGFβRI (Table S2).

## Discussion

**Figure 8:**
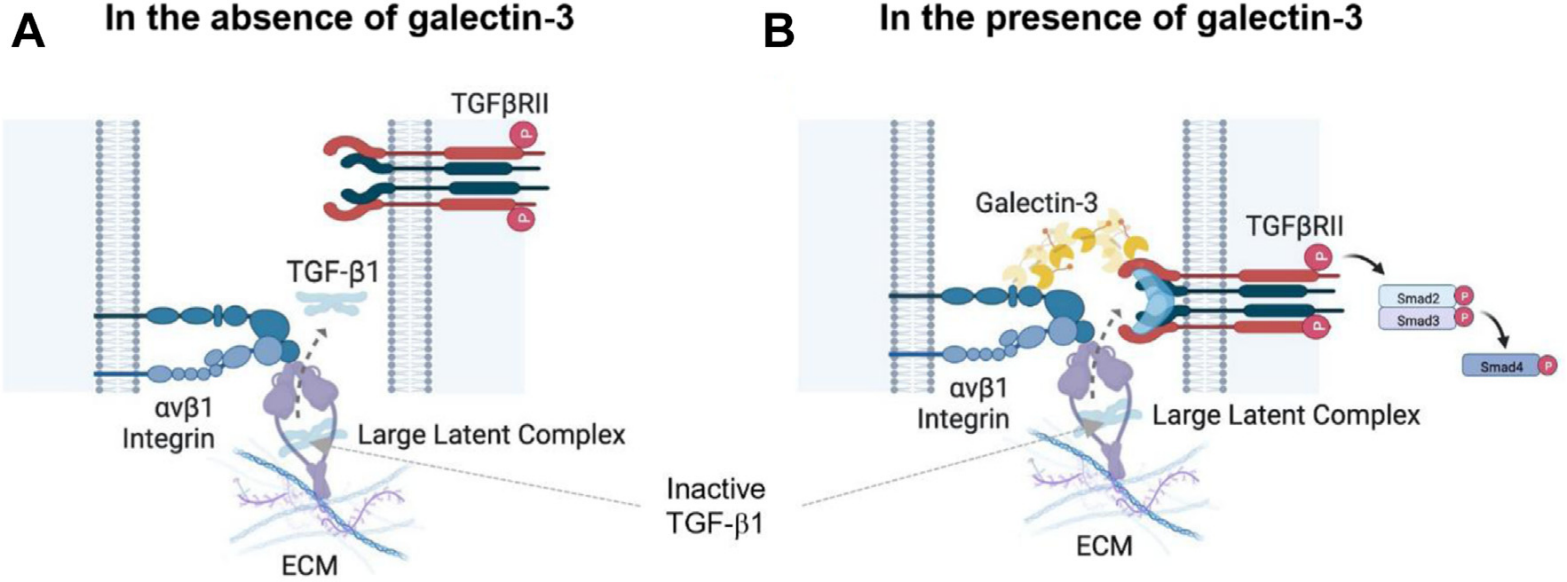
**Diagram of the proposed mechanism of galectin-3–mediated TGF-β1 activation in lung fibroblasts.** Activation of αvβ1 integrins leads to a conformational change, which promotes release of the active TGFβ molecule from the large latent TGFβ complex and triggers downstream signaling following binding to TGF-β1 receptors on neighboring cells. In the absence of galectin-3 (*A*), the αvβ1 integrin and TGF-β1 receptors may not always be found in close proximity on adjacent cells, limiting the degree of TGF-β1 receptor mediated cell signaling initiated. *B*, in the presence of galectin-3, the galectin-3 carbohydrate-binding domain binds to the glycosylation sites on both the αv integrins and the TGF-β1 receptor forming a galectin lattice at the cell surface. This galectin lattice facilitates receptor clustering, bringing the integrin and TGF-β1 receptor into close proximity, and ensuring that the active TGFβ1 molecule can interact with its receptor potentiating TGF-β1 downstream signaling and phosphorylation of Smad2 and Smad3. Galectin inhibitors including GB0139 bind to the galectin-3 carbohydrate recognition domain, block the protein–glycan interactions and prevent galectin lattice–induced clustering. TGF-β1, transforming growth factor-β1.

We show that galectin-3 can activate TGF-β1 and that inhibiting galectin-3 can prevent agonist-induced integrin-mediated TGF-β1 activation in fibroblasts. These data demonstrate that galectin-3 binds integrins and the TGF-β1 receptor in a glycosylation-dependent manner. Galectin-3 binding responses were higher for αvβ1 and αvβ5 than for αvβ6, and this may partially explain the lack of galectin-3–mediated TGF-β1 activation detected in iHBECs despite the expression of high levels of αvβ6 integrin in epithelial cells (39). Although we were only able to detect this mechanism in fibroblasts, we cannot exclude the possibility it may also occur in epithelial cells but that higher concentrations of galectin-3 may be required at the cell surface to initiate the response. We demonstrate that galectin-3 inhibition prevents binding of galectin-3 to integrins, which in turn inhibits TGF-β1 activation in fibroblasts. Given the requirement for the close physical association of the integrin, the latent TGF-β1 complex and TGF-β1 receptor for agonist-induced integrin-mediated TGF-β1 activation (39), we hypothesise that galectin-3 promotes integrin-mediated TGF-β1 activation in lung fibroblasts by facilitating clustering of the β1 integrin and TGF-β1 receptor on their respective cell surfaces (Fig. 8).

Traction-dependent TGF-β1 activation is mediated by the integrins αvβ1, αvβ3, αvβ5, and αvβ6, which directly bind to the latency-associated peptide portion of the latent TGF-β1 complex (19, 21, 40, 41, 42), and the αvβ1 integrin is suggested to be the principal integrin responsible for activation of latent TGF-β1 in fibroblasts (43, 44). The phospholipid LPA has previously been shown to bind its G protein–coupled receptor LPA_2_ to induce αvβ6-mediated TGF-β1 activation in epithelial cells *via* both Ras homolog family member A (RhoA) and Rho-associated protein kinase (45). Although LPA has been shown to mediate primary lung fibroblast chemotaxis and proliferation *via* LPA_1_ following lung injury, the potential contribution of LPA signaling to integrin-mediated TGF-β1 activation in fibroblasts is unexplored (46, 47).

Here, we show evidence of LPA-induced αvβ1-mediated TGF-β1 activation in primary lung fibroblasts and that pretreatment with a β1 integrin inhibitor reduced LPA-induced Smad2 phosphorylation in a concentration-dependent manner. Subsequently, the potential significance of galectin-3 binding to αvβ1 was explored in the context of LPA-induced integrin-mediated TGF-β1 activation *in vitro*. LPA stimulation of HLFs following pretreatment with cell-permeable galectin-3 inhibitors (GB1107 and GB1211) reduced phosphorylated Smad2, reflective of a reduction in TGF-β1 activation levels. As with the β1 integrin, these findings show endogenous galectin-3 as being essential for LPA-induced TGF-β1 activation. More specifically, we confirm that it is the extracellular galectin-3 that is responsible for this mechanism as the cell-impermeable galectin-3 inhibitor (GB0149) (38) also reduced Smad2 phosphorylation in a concentration-dependent manner. This role of extracellular galectin-3 was confirmed in experiments with GB0139 in which the drug completely inhibited TGF-β1 activation as denoted by Smad2 phosphorylation despite being in use for a short incubation time (20 min). During this short time frame, target engagement of intracellular galectin-3 could not occur in response to GB0139 due to poor cell permeability (38), therefore highlighting that the effects on TGF-β1 activation are mediated by extracellular galectin-3.

Galectin-integrin binding by SPR was glycan-dependent, thus supporting the view that extracellular galectin interactions are commonly carbohydrate-dependent (48, 49, 50). Enzymatic removal of all integrin N-linked and common O-linked glycans inhibited the galectin–integrin binding interaction, while only a partial reduction in binding was observed following the removal of N-linked glycans alone. From these experiments, it was not possible to determine whether the partial binding observed following PNGase F treatment was due to O-linked glycan interactions or residual N-linked glycans. Although the efficiency of PNGase F treatment was visually confirmed by a shift in protein mobility by SDS-PAGE, this does not verify 100% N-glycan removal and therefore, some N-glycans may potentially remain on the integrin surface for galectin binding. Alternatively, the O-linked glycans may partially compensate for the higher affinity N-linked glycan interactions, when they are no longer available or there is cooperative binding between the glycan subtypes. These findings are in agreement with the published literature, whereby the carbohydrate dependency of galectin-integrin binding has been demonstrated using a number of techniques other than SPR (35, 37).

The galectin-3 binding responses recorded across all three integrins were considerably higher than the theoretical maximum response for analyte-ligand binding 1:1, demonstrating that multiple individual galectins are involved in the binding interaction. As SPR measures a change in mass, it is not possible to accurately conclude if the total estimated number of galectin-3 protein is directly interacting with the immobilized ligand or if this number is a result of galectin-3 oligomerization. As galectin-3 can oligomerize through either the N-terminal or C-terminal domain, this may have resulted in multivalency of carbohydrate-binding activity, where multivalent galectin-3 binds to one or more cell surface glycans causing high avidity binding (24, 25, 51, 52, 53). Alternatively, each galectin-3 protein could be binding to a single binding site on the integrin as a number of glycosylation sites have been reported or predicated from the NXS/T consensus sequence on the αv (13 sites), β1 (12 sites), β5 (8 sites), and β6 subunits (9 sites) (54, 55). There is currently no consensus sequence motif to enable the prediction and identification of O-glycans. However, it is less likely that each galectin protein is binding to a single glycosylation site given the estimated number of proteins and the size of galectin-3 when compared to the integrin receptor extracellular domain. It is therefore most probable that galectin-3 binds to one or more binding sites on the integrin causing subsequent self-association and lattice formation. Supporting this conclusion, galectin-3 has previously demonstrated positive cooperativity upon binding by SPR (56).

Galectin-3 also bound to the TGFβRII extracellular domain in a glycan-dependent manner. In contrast with integrin–galectin-3 interactions, PNGase F treatment of TGFβRII prevented TGFβRII-galectin-3 binding to a similar level observed with total glycan removal. This suggests that the N-linked oligosaccharides on the extracellular domain of TGFβRII predominantly mediate the galectin-3 binding interaction. This finding is consistent with a previous study from Partridge *et al* (2004) reporting alpha-1,6-mannosylglycoprotein 6-beta-N-acetylglucosaminyltransferase–dependent binding of galectin-3 to the TGFβRII subunit by co-IP (33). In their study, the half-life of the TGF-β1 receptor and subsequent cytokine signaling was reduced in alpha-1,6-mannosylglycoprotein 6-beta-N-acetylglucosaminyltransferase−/− cells, leading to decreased Smad2/3 nuclear translocation in response to TGF-β1 (33). This suggests that N-linked glycans and their subsequent interactions are necessary for TGF-β1 canonical signaling, following TGF-β1 ligand binding. Although it has been confirmed that TGFβRII has two N-linked glycan sites and a third is predicted by sequence analysis (57), as with the integrins, it is not possible to conclude whether galectin-3 binds to all three sites or oligomerizes upon binding. However, our experiments do confirm that protein-glycan binding events occur at the galectin-3 C terminus as small molecule inhibitors of galectin-3 (GB0139 and GB1107), both of which act *via* the galectin CRD, prevented galectin-3 binding to all three integrins and TGFβRII in a concentration-dependent manner.

Using a combination of co-IP and PLAs, we validated our positive SPR data generated with recombinant proteins and confirmed firstly that endogenous galectin-3 binds to the β1 integrin in primary lung fibroblasts and secondly that colocalization of the two proteins (≤40 nm) occurs on the cell surface. In the absence of TGF-β1 stimulation, such colocalization was detectable by PLA in IPF HLFs but not non-IPF cells, suggesting that the proteins are inherently more closely associated in the disease state. The failure to detect a PLA signal in the untreated non-IPF fibroblasts above the limit of detection for this assay may result from a lower level of αvβ1 integrin expression and/or galectin-3 at the cell surface in these cells. Fibroblasts from IPF patients are consistently shown to have increased αvβ1 mRNA expression compared to non-IPF controls (58). Similarly αvβ1 protein levels are increased in IPF lung (59) and galectin-3 expression is known to be increased in fibroblasts within fibrotic foci (28). Exposure of lung fibroblasts to a fibrotic environment, for example, stimulation with TGFβ for 24 h may potentiate both αvβ1 expression and galectin-3 release from on fibroblasts *in vitro* increasing the likelihood that sufficient stochastic galectin-3–αvβ1 interactions are detected by PLA to visualize the signal. Our observations are consistent with nonpeer reviewed results showing that although colocalization of galectin-3 and the β1 integrin by PLA was also not detected in untreated lung *ex vivo* human lung tissue (60), it was detectable in TGF-β1–treated lung tissue samples created using a validated model of lung fibrogenesis (61). Stimulation with TGF-β1 increased the signal in both IPF and non-IPF fibroblasts, although more markedly in the IPF tissue–derived cells, and this response could be entirely abrogated by antagonism of galectin-3 glycan binding using the small molecule inhibitor GB0139. Together these findings support a mechanistic link between TGF-β1 signaling, enhanced complexation of galectin-3 with the β1 integrin *via* CRD:glycan interactions and fibrogenesis in disease. These data are also encouraging given that the concentration of compound used here covers the concentration range used in the phase 1/2a study and is therefore translatable to the clinic (ID: NCT02257177) (32).

Collectively, this work shows that CRD-dependent galectin-3 binding interactions are implicated in TGF-β1 signaling in human lung fibroblasts and that the inhibition of these interactions can modulate TGFβ-dependent signaling and release of fibrogenic mediators in PCLuS from IPF patients. We have performed *ex vivo* validation studies of biophysical data, which confirm that galectin-3 interacts with the β1 integrin in HLFs. We have shown extracellular galectin-3 to be involved in LPA-induced integrin-mediated TGF-β1 activation and alluded to a role for galectin-3 in TGF-β1 signaling at the receptor level. We hypothesize that upon binding, galectin-3 self-associates to form a lattice between the αvβ1 integrin and TGF-β1 receptor on adjacent cells to facilitate receptor clustering. Subsequently, when TGF-β1 is activated *via* traction-dependent mechanisms, it is in close enough proximity to the TGF-β1 receptor to bind and signal, causing fibrogenesis. Understanding the precise role of galectin-3 in IPF pathogenesis may be critical for the continued development of more effective and selective treatments for IPF patients.

## Experimental procedures

### Culture of primary cells

IPF and non-IPF HLFs were obtained from explanted human lung tissue post lung biopsy following informed, written consent, and ethical review (Ethical approval numbers: 08/H0407/1, 20/SC/0142, 10/H0402/12). Briefly, the tissue was washed with PBS and then cut into 1 mm^2^ sections. Two sections were placed per well of a 6-well plate and left to adhere to the plastic for 5 to 10 min before adding culture media. Cells were cultured in Dulbecco's Modified Eagle medium high glucose (Sigma-Aldrich) supplemented with 10% fetal bovine serum (FBS) (Gibco), 4 mM L-glutamine (Sigma-Aldrich), 100 U/ml penicillin, and 100 μg/ml streptomycin (Sigma-Aldrich). Cells were grown in a humidified incubator set to 37 °C with 5% CO_2_. Primary cells were tested for *mycoplasma* and confirmed to be *mycoplasma* free prior to experimentation.

### Culture of immortalized cell lines

iHBECs (gifted from Professor Jerry Shay, University of Texas) were cultured in keratinocyte serum-free media with L-glutamine (Gibco) supplemented with 0.2 ng/ml human recombinant epidermal growth factor (Gibco), 25 μg/ml bovine pituitary extract (Gibco), 25 μg/ml G418 disulphate (VWR), and 250 ng/ml puromycin dihydrochloride (Sigma-Aldrich). Cells were grown in a humidified incubator set to 37 °C with 5% CO_2_. Immortalized cell lines were tested for *mycoplasma* and confirmed to be *mycoplasma* free prior to experimentation.

### Treatment of cells

Cells were growth arrested in serum-free media for 24 h prior to stimulation in the presence or absence of inhibitors. After serum starvation, cells were stimulated with either 10 μg/ml recombinant human galectin-3 protein (R&D Systems) or 2 ng/ml recombinant human TGF-β1 protein (R&D Systems) for 2 h or 50 μM LPA (Sigma-Aldrich) for 4 h.

When used, inhibitors were applied in serum-free media for 20 min prior to stimulation. All of the small molecule glycomimetics utilized in this study, GB0139 (formerly TD139), GB1107, GB1211, or GB0149 were synthesized by the Galecto Biotech AB Medicinal Chemistry Department (Table S1) and used at a concentration range of 0.1 to 10 μM. The ALK5/TGFβRI inhibitor SB-525334 (Sigma-Aldrich) (62, 63) was used at a concentration of 50 μM. The β1 integrin inhibitor NOTT199SS (gifted) was used at a concentration range of 0.1 to 100 nM. All inhibitors were dissolved in 100% dimethyl sulfoxide (DMSO), and all cells, including untreated controls, were exposed to a DMSO concentration equivalent to that used in the highest inhibitor concentration in each experiment.

### Western blot

Protein expression levels of phospho-Smad2 (pSmad2), total Smad2 (tSmad2), and GAPDH were determined by Western blot as previously described (64). Briefly, cells were lysed in protein lysis buffer and protein concentrations determined by Pierce bicinchoninic acid assay (Thermo Fisher Scientific). Protein samples (15–30 μg/lane) were subjected to SDS-PAGE on a 10% SDS-PAGE gel and electroblotted to a polyvinylidene fluoride membrane (Bio-Rad). After blocking for 1 h; 5% nonfat milk in Tris-buffered saline plus 0.1% Tween 20, the membrane was incubated overnight at 4 °C with primary antibody: rabbit anti-pSmad2 mAb (Cell Signaling Technology, Clone No. 138D4; 1:1000), rabbit anti-Smad2/3 pAb (Cell Signaling Technology; 1:1000), or rabbit anti-GAPDH mAb (Abcam, Clone No. EPR16884; 1:10,000). After washes, (Tris-buffered saline plus 0.1% Tween 20) the membrane was incubated for 1 h at room temperature with a horseradish peroxidase (HRP)-conjugated secondary antibody: goat anti-rabbit immunoglobulins/HRP pAb (Dako; 1:3000). All primary and secondary antibodies used were diluted in blocking buffer. The membrane was then incubated with enhanced chemiluminescence (ECL) Western blot detection reagent and visualized by exposing to Hyperfilm-ECL (Thermo Fisher Scientific). Densitometry was performed using ImageJ (www.imageJ.net) and data plotted in GraphPad Prism (www.graphpad.com) as a band density ratio of pSmad2/tSmad2 to determine relative pSmad2 expression.

### Galectin-3 expression and purification for SPR

The human galectin-3 gene sequence was obtained from UniProt (P17931) and delivered as plasmid DNA in a pTwist amp high copy vector (pUC origin of replication derived from pMB1 plasmid, ampicillin resistance; Twist Bioscience). Galectin-3 was cloned into a pOPINF expression plasmid using in-fusion cloning (Addgene plasmid #26042) (65), primers (Sigma-Aldrich) used were:

5′-3′ aagttctgtttcagggcccgATGGCGGATAATTTTAGCTTACATGAC

3′-5′ atggtctagaaagctttaGATCATTGTATAGCTGGCCGAAGTG

Recombination products were used directly for the transformation of competent *Escherichia coli* TOP10 cells (IBA Lifesciences) with carbenicillin selection (50 μg/ml). Plasmid DNA was purified using the QIAprep Spin Miniprep Kit (Qiagen) and sequence verified (Source Bioscience).

Galectin-3 was expressed in Lemo21 (DE3) cells (Thermo Fisher Scientific) in terrific broth media (carbenicillin 50 μg/ml, chloramphenicol 35 μg/ml), inoculated cultures were left to grow (37 °C, 210 rpm) until an *A*_600_ nm of 1.4, cells were induced with 1 mM IPTG and left to grow overnight (22.5 °C, 210 rpm) before harvesting by centrifugation (6239*g*, 13 min). Cell pellets containing galectin-3 were resuspended in lysis buffer; 50 mM Hepes (pH 7.8), 300 mM NaCl, cOmplete EDTA-free protease inhibitor cocktail (1 tablet per 100 ml) (Roche), 0.1 mg/ml lysozyme (Sigma-Aldrich), and 10 μg/ml deoxyribonuclease I (Sigma-Aldrich). Cells were lysed with two passes at 28 kpsi using a cell disruptor (Constant Systems), after which the lysate was clarified (53,343*g*, 30 min). The supernatant containing soluble galectin-3 was purified on a 5 ml HisTrap HP column (Cytiva) pre-equilibrated with buffer containing 50 mM Hepes (pH 7.8), 300 mM NaCl, and 10 mM imidazole. Following binding, the column was washed; 50 mM Hepes (pH 7.8), 300 mM NaCl, and 20 mM imidazole. Bound protein was eluted using an imidazole gradient (start concentration: 0 mM, end concentration: 750 mM) with the protein eluting at approximately 300 mM imidazole. Eluted galectin-3 was dialyzed overnight against 50 mM Hepes (pH 7.8), 300 mM NaCl, 0.3 mM tris(2-carboxyethyl)phosphine. The His-tag was cleaved during dialysis by addition of human rhinovirus 3C-protease at a 1:100 ratio 3C:galectin-3. Cleaved galectin-3 was purified from noncleaved materials using reverse immobilized metal affinity chromatography on a pre-equilibrated 5 ml HisTrap HP column (Cytiva). The FT was collected and the column washed as previous before eluting the bound material; 50 mM Hepes (pH 7.8), 300 mM NaCl, and 375 mM imidazole. Samples of cleaved galectin-3 were purified further using size-exclusion chromatography with a Superdex S75 10/300 column (Cytiva) run at 0.5 ml/min. Fractions of galectin-3 were confirmed using SDS-PAGE analysis, confirming presence and purity. The galectin-3 protein purified for SPR studies was highly pure with minimal contaminant.

### Surface plasmon resonance

All SPR experiments were performed on the Biacore T200 (Cytiva) in running buffer; 0.1 M Hepes (pH 7.4), 1.5 M NaCl, and 0.5% surfactant P20 (HBS-P+ buffer 1×) ±1 mM MnCl_2_. Prior to commencing experiments the instrument was successfully primed and underwent routine normalization to ensure a uniform signal during the assay. Commercially available recombinant human integrins αvβ1, αvβ5, and αvβ6 (R&D Systems) or TGF beta receptor II protein (Abcam) were diluted in 10 mM sodium acetate pH 4 immobilization buffer and immobilized onto a Series S Sensor Chip CM5 (Cytiva) *via* the standard amine coupling protocol. Serial dilutions (2-fold) of galectin-3 in running buffer were injected at a 30 μl/min flow rate, 20 °C, contact time: 120 s and dissociation time: 1200 s (integrins) or 300 s (TGFβRII) into flow cells containing approximately 1000 RU of integrin (equating to approximately 135 Rmax) or approximately 400 RU TGFβRII protein (equating to approximately 251 Rmax). The sensor chip surface was regenerated with 5 mM EDTA for integrin-galectin-3 experiments. All sensorgrams and kinetic plots were baseline-corrected using a flow cell with blank immobilization prior to data analysis (Fig. S1). Binding data was analyzed in GraphPad Prism, if steady state was reached then the K_d_ value (M) and Bmax were determined by nonlinear regression (binding saturation) one-site specific binding. If steady state was not reached, then the minimum number of binding sites was estimated from the response at the highest analyte concentration tested (5000 nM). This was calculated using the following formula (56):

$$\text{Minimum}\;\text{number}\;\text{of}\;\text{binding}\;\text{sites}=\left(\frac{\text{Maximum}\;\text{binding}\;\text{response}\;}{\text{Rmax}}\right)$$

To determine the effects of deglycosylation on galectin-3 binding to integrins or TGFβRII, all glycans were removed from recombinant proteins using protein deglycosylation mix II (New England BioLabs) or N-linked glycans alone removed *via* PNGase F digestion (New England BioLabs) under nondenaturing conditions. Successful deglycosylation was confirmed by SDS-PAGE and deglycosylated proteins immobilized as described above. The same galectin-3 serial dilution was injected across all flow paths at a 30 μl/min flow rate, 20 °C, contact time: 120 s and dissociation time: 600 s (integrins) or 300 s (TGFβRII).

SPR was used to assess the effects of small molecule galectin-3 inhibitors (GB0139 and GB1107) on galectin-3 binding to integrins or TGFβRII. Glycosylated recombinant proteins were immobilized as described above. Galectin-3 inhibitors were serially diluted (2-fold) in running buffer containing 625 nM galectin-3 across the dilution series (stocks of compound in 100% DMSO diluted to a 1.5% final concentration). The serial dilution of compound (+galectin-3) was injected across all flow paths at a 30 μl/min flow rate, 20 °C, contact time: 120 s and dissociation time: 600 s (integrins) or 300 s (TGFβRII). The response values were normalized with respect to the highest RU response. Competition binding curves were analyzed in GraphPad Prism and IC_50_ values determined by nonlinear regression analysis (binding saturation), and specific binding was determined with hill slope.

### Coimmunoprecipitation

Co-IP was performed using the Pierce Crosslink IP Kit (Thermo Fisher Scientific). Cells were harvested by scraping in 1× PBS, pellets were lysed in ice cold IP lysis/wash buffer (300 μl/50 mg wet pellet), and protein concentrations were determined by Pierce bicinchoninic acid assay (Thermo Fisher Scientific). Lysates (650 μg/Co-IP reaction) were precleared using control agarose resin slurry (52 μl/Co-IP reaction) for 1 h at 4 °C. The precleared lysates were then incubated at 4 °C overnight with protein A/G plus agarose coupled and crosslinked to 10 μg of pulldown antibody: mouse anti-integrin beta 1/CD29 mAb (R&D Systems, Clone No. P5D2), rabbit anti-galectin-3 mAb (Abcam, Clone No. EPR19244), mouse IgG1 isotype control mAb (R&D Systems, Clone No. 11711), or rabbit IgG isotype control mAb (Abcam, Clone No. EPR25A). FTs and wash steps were collected until confirmation of successful IP. Elution buffer was added to spin columns and eluates analyzed by Western blot for the presence of antigen. Samples were subjected to SDS-PAGE on a 4 to 12% Bis-Tris gel (Invitrogen) and transferred onto a polyvinylidene fluoride membrane (Invitrogen). After blocking for 2 h; 5% nonfat milk in PBS plus 0.05% Tween 20, the membrane was incubated overnight at 4 °C with primary antibody: rabbit anti-galectin-3 mAb (Abcam, Clone No. EPR19244; 1:1000) or mouse anti-integrin beta 1 mAb (Abcam, Clone No. 12G10; 1:1000). After washes (PBS plus 0.05% Tween 20), the membrane was incubated for 1 h at room temperature with a HRP-conjugated secondary antibody: goat anti-rabbit immunoglobulins/HRP pAb (Dako; 1:3000) or goat anti-mouse IgG/HRP pAb (Invitrogen; 1:5000). All primary and secondary antibodies used were diluted in blocking buffer. The membrane was then incubated with ECL Western blot detection reagent and visualized using chemiluminescent detection.

### Proximity ligation assay

Cells were growth arrested in serum-free media for 24 h prior to experiments. After serum starvation, cells were stimulated with 2 ng/ml recombinant human TGF-β1 protein (R&D Systems) for 24 h and then pretreated with 0.1 to 10 μM galectin-3 inhibitor (GB0139) for 20 min. PLA was performed using the Duolink *In Situ* Red Kit Mouse/Rabbit (Sigma-Aldrich). Prior to PLA, cells were fixed with 4% paraformaldehyde for 10 min and incubated with blocking solution (1× PBS pH 7.4, 10% FBS) for 2 h at 37 °C. Slides were then incubated overnight at 4 °C with primary antibody (5 μg/ml): mouse anti-integrin beta 1 mAb (Abcam, Clone No. P5D2), rabbit anti-galectin-3 pAb (Invitrogen), mouse IgG1 isotype control mAb (Dako, Clone No. DAK-GO1), or rabbit IgG isotype control pAb (BD Biosciences, Clone No. Poly1281). After washes (wash buffer A), slides were incubated for 2 h at 37 °C with PLA probe solution containing anti-rabbit plus and anti-mouse minus probes diluted in antibody diluent (1× PBS pH 7.4, 2% FBS, 1% bovine serum albumin) (1:5). Ligase (1 U/μl) diluted in 1× ligation buffer (1:40) was then added to washed slides (wash buffer A) and incubated for 30 min at 37 °C. Polymerase (10 unit/μl) was diluted in 1× amplification buffer (1:80) and added to slides for 2 h at 37 °C after wash steps (wash buffer A). After washes (wash buffer B), slides were then stained with 4′,6-diamidino-2-phenylindole solution and mounted with fluoroshield for imaging (Leica LSM 980 Airyscan2).

Serial *z*-stack images of individual cells were collected by confocal imaging and data presented as the *z*-stack with maximum intensity projection.

### *Ex vivo* human IPF precision cut lung slice studies

IPF PCLuS experiments were completed as previously described (66). Briefly, human fibrotic lung tissue was sourced ethically from human explants from three patients with IPF undergoing lung transplantation. Tissue was obtained in accordance with local human biological sample management procedures, which were approved by regional ethics approval (11/NE/0291). All patients were male and had their IPF diagnosis confirmed based on medical history and evaluation of their explanted lung tissue by a board-certified respiratory pathologist (IPF donor demographics: age 62, 64, and 65; FEV1 1.85, 2.37, and 1.53; for donors 1, 2, and 3, respectively). Tissue was inflated with 2 to 3% low boiling point agarose and allowed to set at 4 °C. PCLuS were then cut at 400 μm on a vibrating microtome and cultured in small airway epithelial cell growth medium containing bovine pituitary extract (0.004 ml/ml), recombinant human insulin (5 μg/ml), hydrocortisone (0.5 μg/ml), epinephrine (0.5 μg/ml), triiodo-L-thyronine (6.7 ng/ml), recombinant human transferrin (10 μg/ml), retinoic acid (0.1 ng/ml), bovine serum albumin fatty acid free (2.5 mg/ml). PCLuS were rested for 48 h prior to treatment with inhibitors (SB-525334 (ALK5 inhibitor) [Sigma], pirfenidone [Cambridge Biosciences], nintedanib [Cambridge Biosciences], and GB0139 for 4 days with media replaced every 24 h. Soluble mediators released were measured daily using R&D Duoset ELISA kits according to the manufacturer’s protocol. In addition, analysis of experimental proteomics data derived from mass spectrometry testing on the PCLuS at the end of the study (day 6) were completed as previously described (see Supplemental methods). Functional pathway enrichment analysis was performed using the analysis tools at Reactome (https://reactome.org), where upregulated and downregulated proteins from all donors were evaluated for pathway enrichment (see Supplemental methods).

### Statistical analysis

Data analyzed in GraphPad Prism and presented as mean with individual replicates shown.

## Data availability

All data are contained within the manuscript.

## Supporting information

This manuscript contains Supporting information (38, 67, 68, 69, 70).

## Conflict of interests

R. G. J. is a trustee for Action for Pulmonary Fibrosis. A. E. J. is a founder and shareholder of Alevin Therapeutics. J. F. C., F. R. Z., A. C. M., and R. J. S. are Galecto employees with shares/options in the company. N. R. P. is a Roche employee. L. A. B. is a director and shareholder of FibroFind. G. H., R. M. L., P. S., S. B. C., D. J. S., and I. D. S. declare that they have no conflict of interests with the contents of this article.
