# Supplementary material for: Defining the mechanism of galectin-3–mediated TGF-β1 activation and its role in lung fibrosis
Source: J Biol Chem. 2024 Apr 18;300(6):107300. doi: 10.1016/j.jbc.2024.107300 (PMC11134550; doi:10.1016/j.jbc.2024.107300)
Supplement: Supporting Information and Tables S1 and S2 [file mmc1.docx]

**Supporting Information**

**Defining the mechanism of galectin-3-mediated TGF-β1 activation and its role in lung fibrosis.**

Jessica F. Calver^1,2^, Nimesh R. Parmar^1,3^, Gemma Harris^4^, Ryan M. Lithgo^4,5,6,7^, Panayiota Stylianou^8,9^, Fredrik R. Zetterberg^10^, Bibek Gooptu^8,9^, Alison C. Mackinnon^11^, Stephen B. Carr^4,12^, Lee Borthwick^13,14^, David J. Scott^4,5^, Iain Stewart^15^, Robert J. Slack^2^, R. Gisli Jenkins^15^, Alison E. John^15,*^

* a.john@imperial.ac.uk

**Contents :**

Figure S1. SPR Sensorgrams for galectin-3 binding to glycosylated αv integrins or TGFβRII

Table S1. Galectin-1 and Galectin-3 Kd values and cell permeability for Galecto Biotech compounds

Table S2. Pathways affected by protein down-regulation after GB0139 treatment (10 μM) across all IPF patient-derived PCLuS

**Supplemental Methods**

**Mass spectrometry-based proteomics**

*Sample preparation*

Lung slice tissue protein concentration was measured using Pierce™ BCA Protein Assay Kits (Thermo Scientific™) and an equivalent of 100 µg (~2.5 µl) was taken for further processing using S-Trap™ micro spin columns (Protifi). The sample volumes were adjusted to 22.5 µl with S-trap lysis buffer (5% SDS, 50 mM triethylammonium bicarbonate (TEAB) pH 8.5). Proteins were reduced with dithiothreitol at a final concentration of 40 mM (65°C, 30 minutes), cysteines alkylated by incubation with iodoacetamide (80 mM final concentration, 30 minutes, room temperature in dark) and then acidified by adding 27.5 % phosphoric acid to a final concentration of 2.5 % (v/v). The samples were then loaded onto spin columns in 6 volumes of loading buffer (90 % methanol 100 mM TEAB pH 8) and centrifuged at 4,000 *g* for 30 seconds. The columns were then washed with loading buffer (three times) and the flow through discarded. Proteins were digested with trypsin (Worthington) in 50 mM TEAB pH 8.5, at a ratio of 10:1 protein to trypsin, at 47°C for 90 minutes. Peptides were eluted with three washes of the trap; first 50 µl 50 mM TEAB, second 50 µl 0.1 % formic acid and third 50 µl 50 % acetonitrile with 0.1 % formic acid. The solution was frozen then dried in a centrifugal concentrator and reconstituted in 0.1 % formic acid (FA)/2 % acetonitrile (ACN).

*Data Independant Acquisition (DIA)*

Peptide sample (1 µl) was loaded per liquid chromatography–mass spectrometry (LCMS) run, peptides were separated using an UltiMate 3000 RSLCnano HPLC. Samples were first loaded/desalted onto Acclaim PepMap100 C18 LC Column (5 mm Å~ 0.3 mm i.d., 5 μm, 100 Å, Thermo Fisher Scientific) at a flow rate of 10 μl/min^−1^ maintained at 45°C and then separated on 50 cm RP-C18 µPAC™ column (PharmaFluidics) using a 60 minute gradient from 97 % A (0.1 % FA in 3 % DMSO) and 3% B (0.1% FA in 80% ACN 3% DMSO), to 35 % B, at a flow rate of 400 nl/min^−1^. The separated peptides were then injected into Exploris 480 via Thermo Scientific μPAC compatible EasySpray emitter and analysed using data independent acquisition (DIA) at a temperature of 320°C, spray voltage 1,500 V. The total LCMS run time was 90 minutes. Orbitrap full scan resolution was 60,000, RF lens 50 %, Normalised Autogain Control (ACG) Target 300 %, scan range 390-1,600 m/z. DIA MSMS were acquired with 49 variable m/z windows covering 390- 1621m/z, at 30,000 resolution, dynamic maximum injection time with ACG target set to 3,000, and normalized collision energy level of 30%.

*Data processing*

Acquired data was analysed in DIA-NN version 1.8 as previously described (Demichev *et al.*, 2020) against the human proteome database (Uniprot 3AUP00000564-2022.10.20) combined with common Repository of Adventitious Proteins (cRAP), Fragment m/z: 200-1,800, enzyme: Trypsin, allowed missed-cleavages: 2, peptide length: 7-30, precursor m/z 300-1,800, precursor charge: 2-5, Fixed modifications: carbamidomethylation(C), Variable modifications: Oxidation(M).

**Functional pathway enrichment analysis on PCLuS**

MS proteomics data was normalised (using quantile normalisation) and log transformed. Normalised data was analysed in R using the Limma package and all donor data was analysed to compare treatment (GB0139 10 µM) *vs.* vehicle. A P-value threshold equal to 0.05 and a +/- fold-change threshold of 10% shift in protein level was applied. Functional pathway enrichment analysis on the up- and down-regulated proteins was performed using the analysis tools at Reactome (<https://reactome.org>). Reactome is a curated and peer reviewed database which enables map genes/proteins to biologically-functional pathways and provides a measure of statistical enrichment (rather than gene set or gene ontology term enrichment). Using Reactome, up- and down-regulated proteins from all GB0139 treated IPF donors were evaluated for pathway enrichment. Where a pathway was enriched for proteins in this analysis, it could be suggested that this pathway may be functionally perturbed by the change in protein level (whether up or down).

**Figure S1. SPR Sensorgrams for galectin-3 binding to glycosylated αv integrins or TGFβRII**

Baseline-corrected sensorgrams for galectin-3 binding to glycosylated αv integrins: (A) αvβ1, (B) αvβ5 and (C) αvβ6 or (D) the TGFβRII subunit.

**Table S1. Galectin-1 and Galectin-3 Kd values and cell permeability for Galecto Biotech compounds**

| **Compound** | **Galectin-3 K_d_  (µM)** | **CACO-2**  **(A > B/B > A)**  **Papp (10−6 cm/s)** |
| --- | --- | --- |
| GB0139 | 0.0023 | 0.07/0.05 |
| GB1211 | 0.025 | 4.6/30 |
| GB1107 | 0.037 | 15/16 |
| GB0149 | 0.099 | <0.03/<0.07 |

**1** Compound affinity and permeability has previously been assessed for GB0139 (38, 68), GB1211 (69), GB1107 (38, 70) and GB0149 (38, 68).

**Table S2. Pathways affected by protein down-regulation after GB0139 treatment (10 μM) across all IPF patient-derived PCLuS**

**
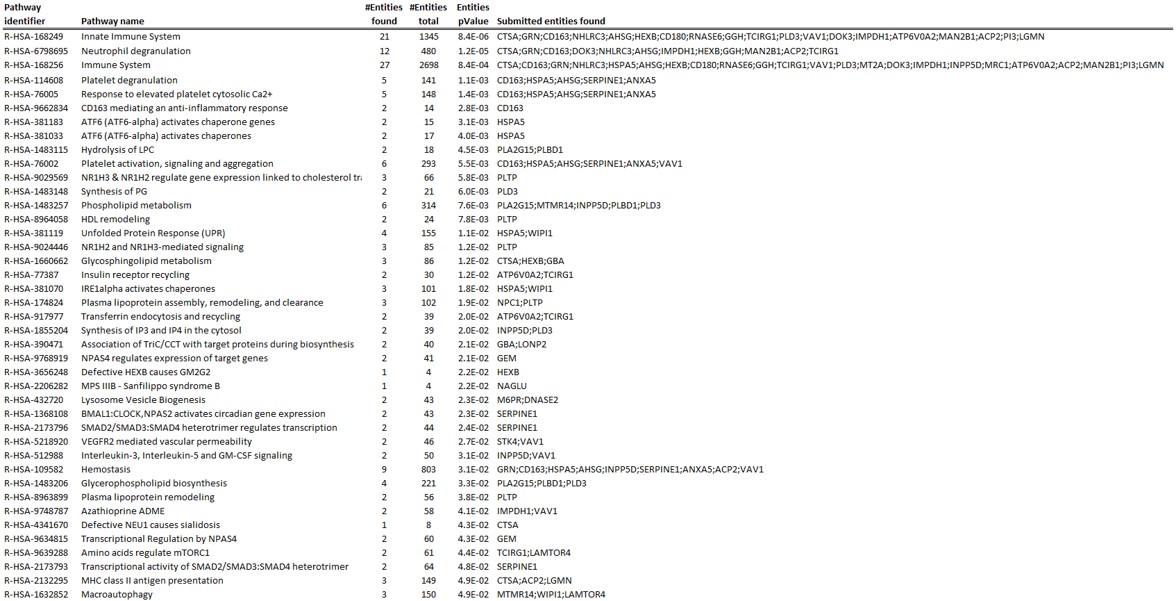
**
